# InTraSeq: A Multimodal Assay that Uncovers New Single-Cell Biology and Regulatory Mechanisms

**DOI:** 10.21203/rs.3.rs-5284652/v1

**Published:** 2024-12-09

**Authors:** Sean Beausoleil, Majd Ariss, Linglin Huang, Xiaokai Ding, Shivani Sheth, Tyler Levy, Jeremy Fisher, Jean Loebelenz, Keith Arlotta, Karen Dixon, Roberto Polakiewicz, Vijay Kuchroo

**Affiliations:** Cell Signaling Technology, Inc., Danvers, Massachusetts, USA; Cell Signaling Technology, Inc., Danvers, Massachusetts, USA; The Gene Lay Institute of Immunology and Inflammation, Brigham and Women’s Hospital, Massachusetts General Hospital and Harvard Medical School, Boston, Massachusetts, USA; Klarman Cell Observatory, Broad Institute of MIT and Harvard, Cambridge, Massachusetts, USA; The Gene Lay Institute of Immunology and Inflammation, Brigham and Women’s Hospital, Massachusetts General Hospital and Harvard Medical School, Boston, Massachusetts, USA; Klarman Cell Observatory, Broad Institute of MIT and Harvard, Cambridge, Massachusetts, USA; Cell Signaling Technology, Inc., Danvers, Massachusetts, USA; Cell Signaling Technology, Inc., Danvers, Massachusetts, USA; Cell Signaling Technology, Inc., Danvers, Massachusetts, USA; Cell Signaling Technology, Inc., Danvers, Massachusetts, USA; Cell Signaling Technology, Inc., Danvers, Massachusetts, USA; The Gene Lay Institute of Immunology and Inflammation, Brigham and Women’s Hospital, Massachusetts General Hospital and Harvard Medical School, Boston, Massachusetts, USA; Klarman Cell Observatory, Broad Institute of MIT and Harvard, Cambridge, Massachusetts, USA; Department of Biomedicine, University of Basel, Basel, Switzerland; Cell Signaling Technology, Inc., Danvers, Massachusetts, USA; The Gene Lay Institute of Immunology and Inflammation, Brigham and Women’s Hospital, Massachusetts General Hospital and Harvard Medical School, Boston, Massachusetts, USA; Klarman Cell Observatory, Broad Institute of MIT and Harvard, Cambridge, Massachusetts, USA

**Keywords:** InTraSeq, single-cell, intracellular protein, post-translational modification, signaling pathway

## Abstract

Single-cell RNA sequencing (scRNA-seq) has revolutionized cell biology by enabling the profiling of transcriptomes at a single-cell resolution, leading to important discoveries that have advanced our understanding of cellular and tissue heterogeneity, developmental trajectories, and disease progression. Despite these important advances, scRNA-seq is limited to measuring the transcriptome providing a partial view of cellular function. To address this limitation, multimodal scRNA-seq assays have emerged, allowing for the simultaneous measurement of RNA expression and protein. Intracellular Transcriptomic and Protein Sequencing (InTraSeq), a novel multimodal scRNA-seq technology described here, enables the concurrent measurement of mRNA, surface markers, cytoplasmic proteins, and nuclear proteins within individual cells through oligo-barcoded antibodies. This method offers a comprehensive approach to studying cellular function by combining RNA and protein pro ling from the same sample and utilizing a relatively simple protocol. The InTraSeq method enables researchers to expand their view of critical intracellular protein expression including post-translational modifications (PTMs) and transcription factors, allowing for the identification of novel cellular subtypes and states that may be obscured by RNA-based analyses alone. This is particularly valuable in understanding the heterogeneity of cell populations and identifying distinct functional states. In this report, we used InTraSeq to characterize the complex cellular states and regulatory mechanisms during Th17 cell differentiation. We simultaneously pro led RNA and protein expression in over 85,000 cells, capturing transcriptional changes, changes in protein expression and the dynamics of signaling pathways at a high resolution. Our results revealed novel insights into Th17 cell differentiation, including the identification of key regulatory factors and their target genes. By simultaneously measuring mRNA, extra and intra-cellular proteins, signaling proteins, and PTMs, InTraSeq offers a comprehensive understanding of cellular processes and enables the identification of novel regulatory mechanisms.

## Introduction

Single-cell RNA sequencing (scRNA-seq) has emerged as a transformative tool in cell biology, enabling the pro ling of transcriptomes at single-cell resolution^1–5^. This technology has revolutionized our understanding of cellular heterogeneity, allowing for the identification of novel cell types, tracking of cell differentiation trajectories, and elucidation of the molecular groundwork of development, disease, and immunity^6–10^. While scRNA-seq offers valuable insights into cellular gene expression^11,12^, achieving a holistic understanding of cellular function necessitates measuring proteins, as they play a substantial role in driving many cellular processes that dictate behavior and phenotype^13^. Additionally, understanding the interplay between RNA expression, protein expression, and post translational modifications (PTMs) is crucial for dissecting complex signal transduction pathways and characterizing the molecular mechanisms behind disease development^14–16^.

Due to the importance of measuring proteins along with mRNA, recent advances in single-cell multimodal assays have emerged as a powerful strategy to tackle the limitations of scRNA-seq. These assays enable the simultaneous measurement of multiple cellular features including RNA expression, protein levels, chromatin accessibility, and epigenetic modifications^17,18^. However, integrating multiple molecular modalities from the same cell presents technical challenges. For instance, capturing both RNA and intracellular protein in single cell datasets often compromises RNA integrity^19,20^ and requires joint embedding with other snRNA-seq datasets to obtain an acceptable single-cell RNA readout^20^. Some multimodal approaches require bridging oligos and are not optimized for cytoplasmic protein readouts^21^. Other methods resort to using nuclei instead of whole cells as their input, resulting in the loss of cytoplasmic proteins and the majority of information from signaling pathways^17,20^.

Additionally, recent studies bypass some of these challenges by constructing separate single-cell libraries for each modality followed by computationally bridging the separate libraries together^18,21^. However, this approach presents additional challenges due to the need for complex computational methods and algorithms and increases the amount of lab benchwork for each additional single cell library all of which introduce opportunities for misinterpretation of results. The computational integration makes the implicit assumption that the two libraries are exact replicates with identical cell compositions, and that the association between chromatin accessibility and gene expression is strong enough to dominate over cell-to-cell heterogeneity. The specificity of these assumptions raises concerns about the quality of the integration and the interpretability of the data.

To address all these limitations and challenges, we introduce InTraSeq (Intracellular protein and Transcriptomic Sequencing), a novel technology with a simple experimental workflow. InTraSeq allows for the simultaneous measurement of RNA, surface markers, cytoplasmic proteins, and nuclear proteins inclusive of PTMs within individual cells, enabling insights into altered protein activity, stability, and expression levels. This capability enables researchers to expand their single cell experiments to include important information about protein expression and post-translational modification typical of signaling molecules at single-cell resolution. Importantly, InTraSeq maintains high-quality measurement of mRNA consistent with current scRNA-seq methods within individual cells without compromising the quality of either data modality. This streamlined experimental workflow eliminates the need to create multiple single-cell multimodal libraries. In addition, the InTraSeq provides us with the ability to integrate activation of intracellular signaling components to the downstream transcriptional activation within the same cell. In this report we demonstrate InTraSeq’s utility to advance our understanding of single-cell biology as exemplified by its application to Th17 T-cell differentiation.

## Results

### InTraSeq generates high-quality single-cell RNA and protein profiles in the same cells

**Figure 1:**
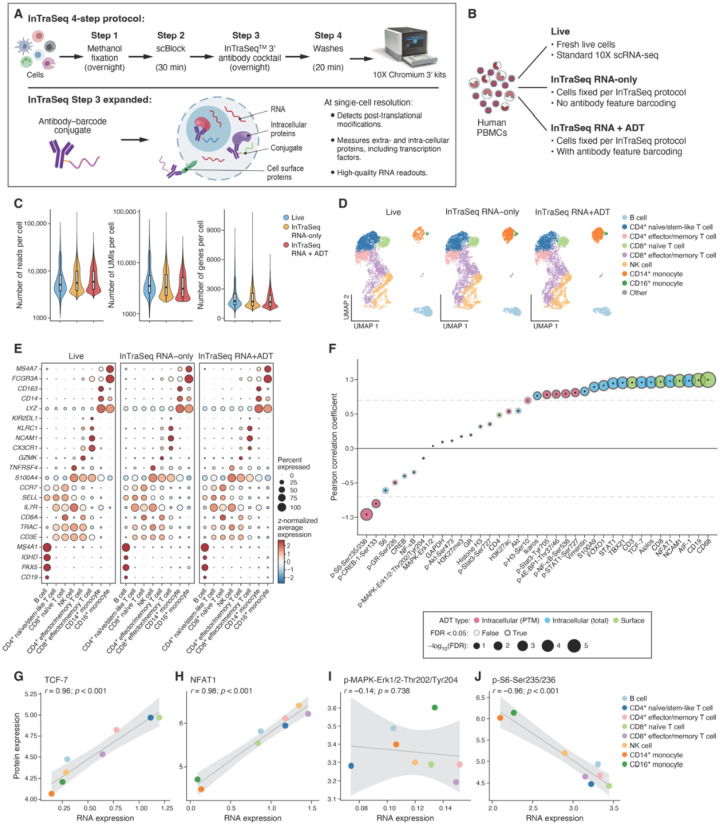
InTraSeq generates high-quality single-cell RNA and protein profiles in the same cells A. InTraSeq workflow. B. Experiment design of the benchmarking analysis. C. Distribution of summary statistics of the gene expression assay comparing the Live, InTraSeq RNA-only and InTraSeq RNA+ADT samples. To exclude impacts of the differences in the sequencing depth and the number of cells captured, only 7,000 cells with largest total number of reads were included in the comparison, and the samples were down-sampled to match the median number of reads per cell in the sample with lowest coverage (Methods). D. UMAP plots depicting the cell types identified in the Live, InTraSeq RNA-only and InTraSeq RNA+ADT samples. UMAP coordinates were computed based on RNA expression data after integration. Colors indicate the cell types. E. RNA expression of cluster marker genes in each sample. Size of dots reflects percent of cells with non-zero UMIs, and the color represents average expression of genes that were z-normalized across cell types in each sample. F. Correlation between average RNA and protein expression across cell types in the InTraSeq RNA+ADT data. Size and border color of dots reflect the statistical significance of the Pearson correlation coefficient. Fill color of the dots shows the protein types. G-J. Average RNA (x-axis) vs. protein (y-axis) expression across cell types for TCF-7 (G), NFAT1 (H), p-MAPK-ERK1/2-Thr202/204 (I) and p-S6-Ser235/236 (J). Pearson correlation coe cients and corresponding unadjusted p-values (using the Algorithm AS 89^49^) were reported for each RNA-protein pair.

The InTraSeq 3’ assay concurrently profiles the transcriptome and proteome in the same cells at single-cell resolution. InTraSeq uses a buffer kit which processes the cells for a total of one hour of benchwork spread out over three days (Fig. 1A, Supplementary Protocol 1). First, the cells are fixed with Methanol overnight. At this point the cells can be stored for up to 7 days at −20C°. After fixation, the cells are incubated with the “scBlock” buffer (Cat #82906) and then immunostained with antibody oligo conjugates for 16 hours. On the third day, the cells were washed three time with a Wash Buffer before being subjected to a scRNA-seq using the10x Genomics 3’ assay (Cat#CG000317). In order to effectively enable antibody capture without interfering with mRNA capture, we leveraged the 10x Genomics Feature Barcoding technology in the oligo design (see methods).

To demonstrate whether InTraSeq can generate high-quality single-cell mRNA profiles, the transcriptomes in human peripheral blood mononuclear cells (PBMCs) from the same donor were benchmarked by performing a standard 10x Genomics Chromium Single Cell 3’ v3.1 experiment in the following three conditions: 1- live PBMCs (Live), 2- PBMCs that underwent the InTraSeq 3’ protocol withoutthe addition of antibody feature barcodes (InTraSeq RNA-only), and 3- PBMCs that underwent the InTraSeq 3’ protocol with antibody feature barcodes (InTraSeq RNA + ADT) (Fig. 1B). After matching on the mean reads per cell for the top 7,000 cells with highest total UMI counts, the InTraSeq RNA-only and InTraSeq RNA + ADT samples displayed similar total UMIs per cell and number of genes per cell compared to the Live PBMCs sample (Fig. 1C, Supplementary table 2, Methods).

Deeper analysis of the RNA profiles shows that RNA expression levels were tightly correlated between the InTraSeq samples and the live control (Extended Data Fig. 1A). The three samples were integrated using the standard Harmony integration pipeline with default parameters^22^ (Extended Data Fig. 1B). Unsupervised clustering analysis showed the three datasets have similar cell type compositions, and the cell type relative frequencies are consistent with the previously published experiments^23^ (Fig. 1D, Extended Data Fig. 1C, Supplementary Table 3). Similar expression patterns of cell type marker genes across datasets were also observed, such as *CD19, CD3E, CD8A, SELL, NCAM1, CD14*, and *FCGR3A*, further validating high delity in mRNA expression between InTraSeq and 10x scRNA-seq data (Fig. 1E). Replication of this experiment yielded consistent results across three biological replicates (Extended Data Fig. 1D-F).

Furthermore, the RNA and protein expression in the InTraSeq RNA + ADT data were compared at the cell type level. As expected, significantly positive correlations between RNA and protein abundance were observed for the many of the targets, particularly the surface proteins such as Macrosialin (CD68), B-lymphocyte antigen CD19 (CD19), Neural cell adhesion molecule 1 (NCAM1) and T-cell surface glycoprotein CD8 alpha chain (CD8a), and some intracellular proteins such as Allograft inflammatory factor 1 (AIF-1), Nuclear factor of activated T-cells, cytoplasmic 2 (NFAT1), and Transcription factor 7 (TCF-7) (Fig. 1F–H). However, the Pearson correlation coefficient rapidly decreased for many intracellular proteins such as Glucocorticoid Receptor (GR), Mitogen-activated protein kinase 3 and Mitogen-activated protein kinase 1 (MAPK-ERK1/2), Cyclic AMP-responsive element-binding protein 1 (CREB-1) (|r| < 0.5, FDR^24^ ≥ 0.05). Additionally, 10 out of 14 of the antibodies against post-translational modifications (PTMs) tended to exhibit minimal (FDR ≥ 0.05) or even negative correlations with the expression of their encoding genes (Fig. 1F, I, J). This was not surprising since PTMs are regulated at the protein levels and cannot be inferred from RNA expression alone. This highlights the unique advantage of InTraSeq, which enables the measurement of PTMs at the single-cell level, particularly when high quality PTM specific antibodies are available providing novel insights that are inaccessible through scRNA sequencing alone.

### InTraSeq captures extra- and intra-cellular protein signals to distinguish cell types and reveal cell states

**Figure 2:**
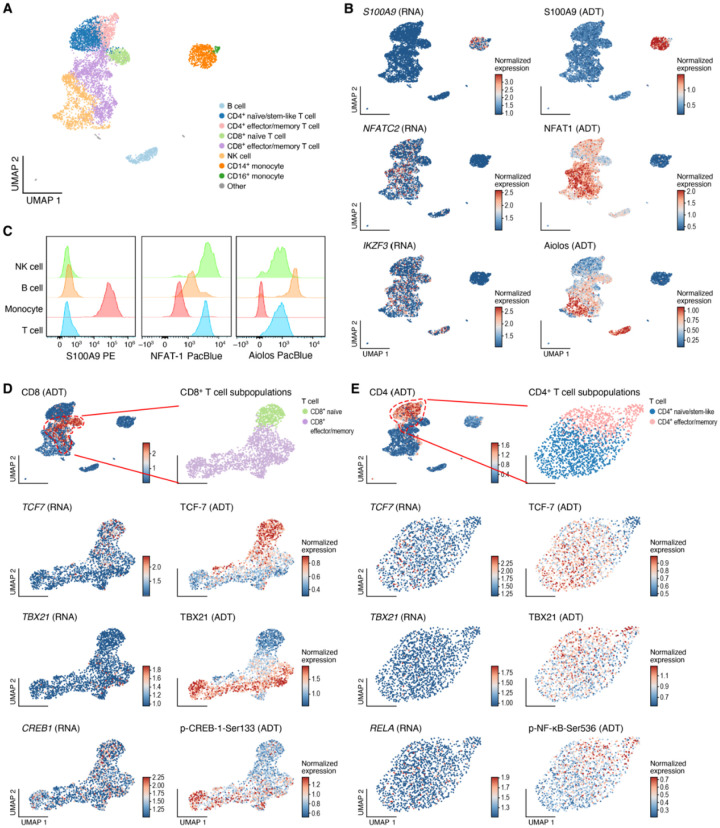
InTraSeq captures extra- and intra-cellular protein signals to distinguish cell types and unveil intricate cell states A. UMAP plot depicting the cell types in the InTraSeq RNA+ADT sample. UMAP coordinates were computed based on RNA expression data of the sample. Colors indicate the cell types. B. UMAP plots depicting the expression of genes of interest (RNAs) and their corresponding proteins (ADTs). The RNA data were log-normalized within every cell, and the ADT data were central-log-ratio-normalized across all cells. C. Flow cytometry validation of the expression patterns across cell types for proteins of interest. D. UMAP plots showing the CD8+ T cell states and the expression of genes and proteins of interest. The top-left panel showed the CD8 protein expression and the location of CD8+ T cell clusters in the whole InTraSeq RNA+ADT data. The top-right panel showed the two CD8+ T cell subpopulations. The UMAP coordinates for all panels other than top-left were computed based on RNA data of the two CD8+ T cell clusters. E. UMAP plots showing the CD4+ T cell states and the expression of genes and proteins of interest. The top-left panel showed the CD4 protein expression and the location of CD4+ T cell clusters in the whole InTraSeq RNA+ADT data. The top-right panel showed the two CD4+ T cell subpopulations. The UMAP coordinates for all panels other than top-left were computed based on RNA data of the two CD4+ T cell clusters.

To assess the quality and robustness of the InTraSeq protein signal, a deeper analysis of the InTraSeq RNA + ADT sample was performed. As demonstrated in Fig. 1, the high-quality mRNA expression data itself allowed the identification of different cell types (Fig. 2A). While many of the protein and mRNA expression patterns were consistent in feature plots and observed in the same cluster of cells in the UMAP, the protein data exhibited less sparsity compared to the RNA data (Fig. 1F, Fig. 2B, Extended Data Fig. 1B-C, and Supplementary Table 4). To validate the InTraSeq protein signal of Protein S100-A9 (S100A9), NFAT1, and Zinc finger protein Aiolos (Aiolos), an independent flow cytometry analysis was conducted in a separate PBMC sample. The flow results were similar to the InTraSeq dataset showing S100A9 to be expressed in monocytes, NFAT1 being predominantly observed in T and NK cells with low expression in B cells, and Aiolos being predominantly expressed in B cells then NK and T cells (Fig. 2C). The cross-validation using flow cytometry demonstrates the reliability of the InTraSeq single-cell protein data.

To determine whether InTraSeq’s intracellular protein readout can reveal novel biological insights, mRNA and protein expression were analyzed in different cellular states of one cell type. A CD8 + T cell focused analysis was performed to compare two CD8 + cellular states: a “naïve” population characterized by high *CCR7*, and *SELL* transcript expression and an “effector/memory” population defined by high *S100A4* and *GZMK* transcript levels^25,26^ (Fig. 1E). TCF-7, a known protein marker of T cell stemness, and T-box transcription factor TBX21 (TBX21), a known transcription factor associated with effector/memory T cells exhibited distinct protein expression patterns in the “naïve/stem” and “effector/memory” cell populations respectively on the UMAP, aligning with previous studies^27,28^ (Fig. 2D). Additionally, phosphorylation of CREB-1 on Ser133 was enriched in effector/memory CD8 + T cells, consistent with its role in T cell activation^29^ (Fig. 2D). In contrast, RNA expression of *TCF7* and *TBX21* was sparse and lacked clear differentiation across CD8 + T cell states (Extended Data Fig. 2D), reinforcing the utility of both protein and PTM readouts to effectively de ne differential cell states.

A similar analysis of CD4 + T cells revealed analogous protein expression patterns for TCF-7 and TBX21 in line with previous studies^27,30^, with corresponding RNA data showing sparser signals (Fig. 2E and Extended Data Fig. 2E). Furthermore, phosphorylation of Transcription factor p65 on Ser536 (p-NF-κB-Ser536), linked to T cell activation^31^, was enriched in effector CD4 + T cells at the protein level but not at the RNA level (Fig. 2E and Extended Data Fig. 2E).

These findings demonstrate InTraSeq’s ability to detect robust proteomic signals, enabling deeper exploration of cellular heterogeneity and identification of cell states, particularly for genes with low RNA expression or proteins regulated by post-translational modifications.

### InTraSeq reveals transcriptomic and proteomic dynamics during Th17 cell differentiation

In addition to characterizing cell states, InTraSeq’s simultaneous measurement of intracellular proteins, post translational modifications, and transcripts facilitates the investigation of signaling pathway activation and subsequent transcriptional changes upon stimulation at a single-cell resolution. To demonstrate this, the Th17 cell differentiation was studied using InTraSeq.

**Figure 3:**
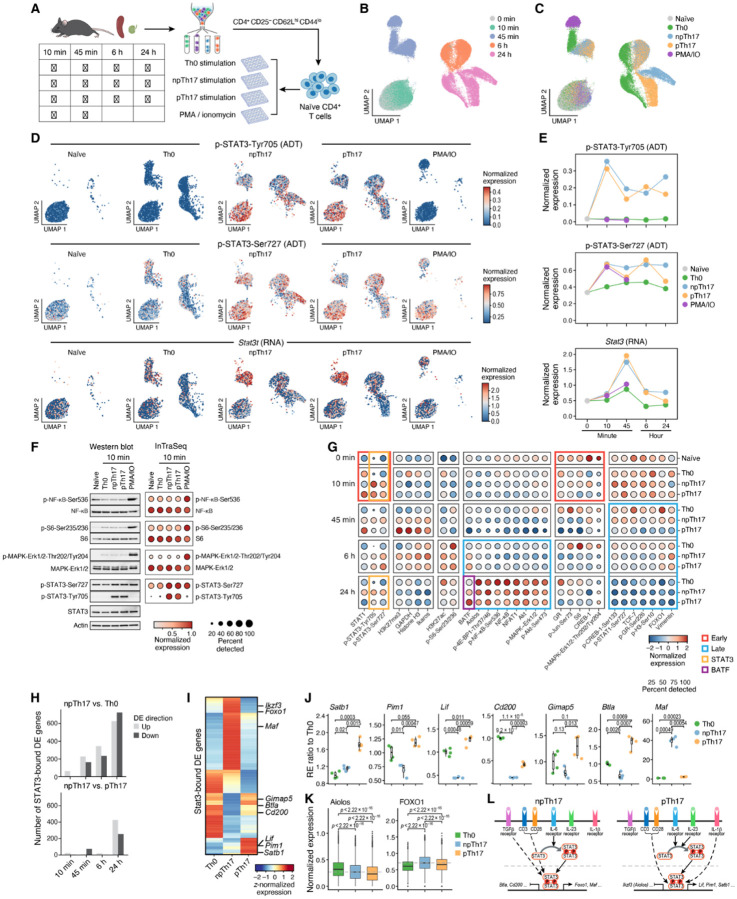
InTraSeq captures the proteomics signals and transcriptional changes during Th17 cell differentiation A. Schematic of experiment design. B. UMAP plot showing the cells of different time points. UMAP coordinates were computed using RNA data. C. UMAP plot showing the cells of different stimulations. The UMAP coordinates are the same as Figure 3B. D. UMAP plots showing the p-STAT3-Ty705 protein, the p-STAT3-Ser727 protein, and *Stat3*RNA expression in each stimulation. The UMAP coordinates are the same as Figure 3B. E. Average expression of p-STAT3-Ty705 protein, the p-STAT3-Ser727 protein, and *Stat3*RNA in every condition over time. F. A comparison of the Western blot validation results (left) and InTraSeq data (right) for selected proteins showed substantial change at 10 minutes compared to naïve CD4+ T cells. G. Dot plot depicting the expression of all proteins at every time and stimulation combination except PMA/IO treatment. Proteins were ordered and clustered using hierarchical clustering. H. Number of STAT3-bound genes that are significantly up- or down-regulated in npTh17 compared to Th0 (top) or pTh17 (bottom) at each time point. I. Expression of STAT3 direct targets of interest at the 24-hour time point. The color indicates the z-normalized average expression. J. qPCR validations of selected STAT3 direct targets of interest. Y-axis represents the ratio of a sample’s relative expression (RE) compared to the mean RE of the Th0 group. The unadjusted p-values were computed using Wilcoxon rank-sum tests. K. Expression of STAT3 direct targets of interest that were included in the InTraSeq antibody panel. The unadjusted p-values were computed using Wilcoxon rank-sum tests. L. Schematic of non-pathogenic and pathogenic Th17 differentiation pathways according to the STAT3-focused analysis.

Previous studies have shown that the Th17 cell differentiation pathway activation occurs within minutes of stimulation^32^, while the transcriptional pro le changes initiate within hours and evolves over days^33^. To capture the dynamics of both the signaling pathways and the gene expression, naïve CD4 + T cells from mouse spleens and lymph nodes were cultured under Th0 differentiation conditions (anti-CD3 + anti-CD28), non-pathogenic Th17 differentiation conditions (npTh17; anti-CD3 + anti-CD28 + Interleukin-6 (IL-6) + Transforming growth factor beta-1 proprotein (TGF-β1)), pathogenic Th17 differentiation conditions (pTh17; anti-CD3 + anti-CD28 + IL-6 + Interleukin-1 beta (IL-1β) + Interleukin 23 (IL-23)) or PMA and Ionomycin (PMA/IO) stimulation (anti-CD3 + anti-CD28 + PMA + Ionomycin). Cells were collected at 0 minutes (naïve cells), 10 minutes, 45 minutes, 6 hours and 24 hours post-stimulations (Fig. 3A). The PMA/IO stimulation condition served as a positive control since they potently activate multiple signaling cascades bypassing the need for TCR engagement and costimulatory signals, with data collected at 10 and 45 minutes.

The InTraSeq Th17 dataset profiled RNA and protein expression in 83,772cells across 16 samples (4,243–6,811 cells per sample, Supplementary Table 5). Unsupervised clustering analysis based on mRNA data revealed distinct clusters from different timepoints, with transcriptional differences between Th0 and Th17 differentiation conditions emerging at 45 minutes (Fig. 3B, Extended Data Fig. 3A-B). The pTh17 and npTh17 conditions were transcriptionally distinct only at 24 hours, indicating delayed cytokine effects on the pathogenic state driven by IL-1β and IL-23, and the non-pathogenic states driven by TGF-β1 (Fig. 3C, Extended Data Fig. 3A-B).

Additionally, the 10-minute post-stimulation samples did not cluster separately from the naïve CD4 + T cells since the differences in the transcription profiles were insufficient to drive distinct clusters at this early timepoint (Fig. 3B, Extended Data Fig. 3A-B). In contrast, protein-based single-cell clustering demonstrated pronounced clustering difference at 10 minutes between naïve and differentiated cells, highlighting InTraSeq’s ability to quantify early proteomic changes not observable using RNA alone (Extended Data Fig. 3C-F). The contribution of the abundance of PTM-specific antibodies in InTraSeq enabled the detection and visualization of early acute proteomic changes, revealing novel insights into complex cellular dynamics prior to the activation of the transcriptional machinery.

Previous studies have emphasized the critical role of Signal transducer and activator of transcription 3 (STAT3) in Th17 differentiation. This process is initiated by IL-6 binding to its receptor, IL-6 receptor (IL-6R), which subsequently activates Tyrosine-protein kinase JAK1 (JAK1) and Tyrosine-protein kinase JAK2 (JAK2) kinases. These activated kinases then phosphorylate STAT3, triggering its dimerization and translocation to the nucleus where it induces the transcription of genes involved in Th17 cell differentiation^34,35^. The time course InTraSeq data enabled observation of dynamic changes in phosphorylation of STAT3 on Tyr705 or Ser727 (p-STAT3-Tyr705, p-STAT3-Ser727), and *Stat3* mRNA relative to naïve and Th0 samples (Fig. 3D–E). Consistent with previous findings, p-STAT3-Tyr705 levels significantly increased and peaked in npTh17 and pTh17 conditions as early as 10 minutes after stimulation, persisting at a slightly lower level until 24 hours. While p-STAT3-Ser727 also increased at 10 minutes, this change was observed in PMA/IO stimulation, suggesting involvement of mechanisms beyond the IL-6 signaling pathway, such as TCR activation. *Stat3* RNA increased at 45 minutes, with highest levels in npTh17 and pTh17 conditions, followed by a sharp decline at 6 hours. The distinct temporal patterns of p-STAT3 protein and RNA levels support its known positive autoregulatory role, wherein STAT3 activation in CD4 + T cells drives its own expression by binding to the *Stat3* gene promoter^36^.

To validate the p-STAT3 signal, Western blot analysis was performed on naïve T cells and stimulated Th0, npTh17, pTh17, and PMA/IO cells collected 10 minutes post-stimulation. Consistent with InTraSeq protein data, p-STAT3-Tyr705 upregulation was observed exclusively in Th17 samples (Fig. 3F). Similarly, p-NF-κB-Ser536, phosphorylation of Small ribosomal subunit protein eS6 on Ser235/236 (p-S6-Ser235/236), and phosphorylation of MAPK-ERK1/2 on Thr202/204 (p-MAPK-ERK1/2-Thr202/204) were upregulated in PMA/IO samples by both Western blot and InTraSeq analysis(Fig. 3F). Cross-validation with Western blot supports the robustness of the InTraSeq single-cell phosphoprotein data, and demonstrates the ability to simultaneously measure mRNA, protein, and PTM expression in a single experiment.

Furthermore, additional InTraSeq protein readouts in the Th17 experiment revealed diverse patterns across conditions and time points (Fig. 3G). Notably, activation and repression modules were identified based on protein expression dynamics. For example, Forkhead box protein O1 (FOXO1) and TCF-7 transcription factor protein levels decreased at the 45 minutes timepoint, with a substantial drop in the Th17 stimulation conditions relative to Th0 (Fig. 3G, Extended Data Fig. 3G). Conversely, Basic leucine zipper transcriptional factor ATF-like (BATF) and Aiolos both increased after 24 hours. However, BATF showed a larger increase in pTh17 compared to npTh17 and Th0 at 24 hours, while Aiolos had the highest increase in the Th0 sample at the same time point (Fig. 3G, Extended Data Fig. 3H-I).

To elucidate regulatory mechanisms in Th17 cells, STAT3 and BATF were selected as example targets (Fig. 3H–K and Extended Data Fig. 3K-N). Given p-STAT3-Tyr705 upregulation in Th17 differentiation, direct STAT3 target genes were identified using published STAT3 ChIP-seq data from npTh17 cells to determine STAT3-bound gene signatures^37^ (Methods; Supplementary Table 8). The number of STAT3-bound genes that are differentially expressed in npTh17 vs. Th0 gradually increased at each timepoint, peaking at 24h, with 628 upregulated genes and 724 downregulated genes (FDR < 0.05, fold change > 1.5; Supplementary Table 9), demonstrating the impact of STAT3 in the Th17 differentiation system (Fig. 3H).

Additionally, the InTraSeq experiment also showed decreased p-STAT3 levels in pTh17 relative to npTh17 at 24 hours (Fig. 3E). To further dissect this, a deeper analysis was performed to determine whether the STAT3-bound genes are differentially expressed between the npTh17 and pTh17 samples. The results show a striking upregulation of 426 and downregulation of 225 STAT3-bound genes exclusively at the 24h timepoint (Fig. 3H; FDR < 0.05, fold change > 1.5; Supplementary Table 9).

To identify STAT3 direct targets that play a role in Th17 differentiation and associated with Th17 pathogenicity, STAT3-bound npTh17 vs. Th0 differential genes (Fig. 3H, top) were intersected with STAT3-bound npTh17 vs. pTh17 differential genes (Fig. 3H, bottom).

These STAT3 direct targets of interest were differentially regulated by p-STAT3 signaling perturbations in non-pathogenic and pathogenic Th17 conditions (Fig. 3I). For instance, robust transcriptional changes occurred with *Satb1, Pim1, Lif, Cd200, Gimap5*, and *Btla* being upregulated in pTh17 compared to npTh17, and *Maf* being downregulated, with the data being further validated using qPCR (Fig. 3I–J, Fig. 3L).

To further elucidate the p-STAT3 signaling mechanism in the pathogenicity of Th17 cells, two direct targets of STAT3, *Ikzf3* (encoding gene for the Aiolos protein) and *Foxo1* (encoding gene for the FOXO1protein) were found to be significantly downregulated at the transcript level in pTh17 relative to npTh17 at 24 hours (Fig. 3I). Given that antibodies against Aiolos and FOXO1 are in the InTraSeq antibody panel, the protein expression levels of these two targets were compared across conditions at the 24-hour timepoint. Consistent with the mRNA data, the InTraSeq protein levels for Aiolos and FOXO1were also downregulated in pTh17 (Fig. 3K), supporting the differential expression of these direct STAT3-bound genes. This finding underscores the ability of InTraSeq to deconvolute subtle differences in signaling pathways, such as the downregulation of Aiolos and FOXO1in pTh17 compared to npTh17, which is driven by altered STAT3 signaling and supported by both mRNA and protein data (Fig. 3L).

A similar analysis was performed focusing on BATF, given its late activation and differential expression between pTh17 and npTh17 conditions (Fig. 3G). Using publicly available ChIP-seq data^37^, BATF-bound target genes were identified and a subset of the genes were found to be differentially expressed in npTh17 vs. Th0 and/or pTh17 at the 24-hour time point (Extended Data Fig. 3M-N; Supplementary Table 10).

### InTraSeq allowed identification of mitotic cells using phosphorylated Histone H3 (Ser10) abundance

**Figure 4:**
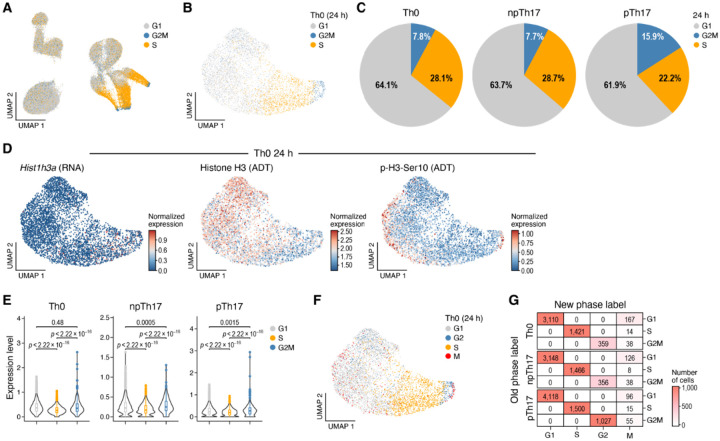
InTraSeq identifies mitotic cells with post-translational modification of Histone H3 A. UMAP plot showing the cell phase labels for all samples based on RNA data. UMAP coordinates were the same as Figure 3B. B. UMAP plot showing the cell phase labels re-annotated for the Th0 stimulation sample at 24 hours. Re-annotation and UMAP coordinates were computed based on the RNA data of the Th0 at 24-hour data. C. Pie chart showing the proportion of cells belong to every cell cycle phases after re-annotation. D. UMAP plots showing the expression of *Hist1h3a* gene (RNA), total Histone H3 protein, and p-H3-Ser10 protein in Th0 at 24 hours. E. Distribution of p-H3-Ser10 protein levels in cells at different cell cycle phases at 24 hours. The unadjusted p-values were computed using Wilcoxon rank-sum tests. F. UMAP plot showing the new cell cycle phase annotation using additional information from p-H3-Ser10 protein levels in Th0 at 24 hours. G. A comparison of the old and new cell cycle phase labels in each sample at 24 hours. The numbers are the number of cells belong to the category.

Cell cycle regulation is crucial for immune cell differentiation and expansion. Performing a default cell cycle scoring based analysis on the single cell mRNA data identified proliferating cells (S, G2, and M phase) (Methods) primarily at the 24 hours timepoint in the Th17 experiment, consistent with expected cell cycle synchronization following prolonged cell culturing (Fig. 4A). To re ne cell cycle phase distribution, cell cycle signature scores were recalculated respectively for every sample at 24 hours, revealing 7.8%−15.9% of cells in S phase, 61.9%−64.1% of cells in G1 phase, and 22.2%−28.7% of cells in G2M phase (Fig. 4B–C). Histone H3 modifications are linked to dynamic chromatin and cell cycle progression^38^. Specifically, phosphorylation of Histone H3 on Ser10 (p-H3-Ser10) marks chromosome condensation during mitosis^39^. To investigate cell cycle dynamics, RNA expression of Histone H3 encoding gene (*Hist1h3a*), total Histone H3 protein, p-H3-Ser10, and the p-H3-Ser10 to total Histone H3 protein ratio were examined (Fig. 4D, Extended Data Fig. 4A-B). While *Hist1h3a* expression significantly correlated with S and G2M gene signature scores (Pearson *r*_*S*_ ∈ (0.24, 0.31) and *r*_*G2M*_ ∈ (0.21, 0.25), respectively; all p-values < 2.2×10^− 16^), total Histone H3 protein levels showed negative correlations (Pearson *r*_*S*_ ∈ (−0.43, −0.34) and *r*_*G2M*_ ∈ (−0.38, −0.22), respectively; all p-values < 2.2×10^− 16^). p-H3-Ser10 (and its ratio to H3) was enriched in a subset of the G2M and G1 phase cells (Fig. 4D–E, Extended Data Figure S4A-C). This discrepancy between RNA, protein, and PTM data suggested limitations in assessing cell cycle states using mRNA alone. To address this, cells with high p-H3-Ser10 levels were annotated as mitosis (M) phase cells (Methods). This approach identifies 4.3%, 3.3% and 2.4% of cells as M phase cells in Th0, npTh17 and pTh17 samples respectively at the 24-hour timepoint (Fig. 4F–G, Extended Data Fig. 4D-E), demonstrating InTraSeq’s sensitivity in capturing transient cell cycle phases. The relatively low percentage of cells in the M phase (4.3%−7.8%) is consistent with the shorter duration of mitosis compared to interphase (G1, S, and G2 phases)^40^. This finding underscores the importance of InTraSeq for accurately capturing transient cell states that may be overlooked by mRNA-based analyses alone.

## Discussion

This study demonstrates the effectiveness of InTraSeq for comprehensive RNA and protein pro ling in both human and mouse cells, offering valuable insights into cellular biology. The PBMC dataset showcased accurate and robust mRNA and protein measurements, further validated by flow cytometry. The use of PBMC cell types as internal controls ensured the specificity and sensitivity of antibody-based protein detection in InTraSeq.

InTraSeq’s ability to measure transcription factors represents a significant advancement over traditional single-cell mRNA sequencing methods. Transcription factor (TF) transcripts are notoriously difficult to detect in single-cell datasets due to their low abundance and rapid turnover^41^. InTraSeq addresses this limitation by enabling the measurement of key TFs such as TCF-7, NFAT1, TBX21, and Aiolos. This capability provides valuable insights into cellular differentiation and function, as TFs play pivotal roles in regulating gene expression and cellular identity.

In circumstances where mRNA and protein expression patterns diverge, InTraSeq excels in identifying and characterizing cellular states that were often overlooked in traditional mRNA-based analyses. For instance, InTraSeq robustly labeled CD4/CD8 states, enabling the characterization of subpopulations within one de ned cell type (Fig. 2D–E). This capability is particularly valuable in cancer biology, where malignant cells may exhibit diverse states not readily characterized by mRNA expression alone.

This study applied InTraSeq to profile RNA and protein expression during CD4 + Th17 cell differentiation, capturing signaling activities through intracellular protein analysis, particularly PTMs, prior to significant transcriptional changes. Using STAT3 as an example, we demonstrated InTraSeq’s ability to identify signaling and transcriptional regulatory pathways, focusing on early-stage (within 24 hours) non-pathogenic and pathogenic Th17 differentiation. Despite the established role of STAT3 in Th17 differentiation, novel STAT3 target genes potentially influencing Th17 pathogenicity were identified.

Furthermore, InTraSeq’s ability to identify novel regulatory mechanisms is exemplified by the discovery of a potential role for BATF in Th17 differentiation. BATF is a transcription factor known to be involved in T cell differentiation, but its specific role in Th17 cells is not well understood. By analyzing protein expression dynamics and identifying BATF target genes, InTraSeq revealed a novel role for BATF in regulating specific gene expression programs associated with Th17 pathogenicity.

InTraSeq’s ability to measure acute proteomic changes at the post-translational modification level, even within short timeframes where RNA expression remains relatively unchanged, underscores its value in studying rapid cellular perturbations. For example, the Th17 cell differentiation experiment demonstrated InTraSeq’s ability to quantify p-STAT3-Tyr705, p-STAT3-Ser727, p-MAPK-ERK1/2-Thr202/204, p-S6-Ser235/236, and p-NF-κB-Ser536 at 10 minutes, a time point where RNA changes were minimal (Fig. 3E, 3G). This highlights the importance of InTraSeq for detecting early-stage signaling pathway activities and cellular responses that precede transcriptional alterations.

While the study provides valuable insights, it has limitations. The Th17 differentiation data were derived from pooled cells without cell hashing or repeated experiments, affecting statistical analysis. To address this, findings were validated using Western blot and qPCR. Future studies could benefit from incorporating cell hashing and biological replicates to enhance statistical rigor. Additionally, expanding the antibody panel in InTraSeq would enable a more comprehensive analysis of Th17 cell states, such as the inclusion of markers specific to pathogenic and non-pathogenic Th17 differentiation.

In the STAT3 and BATF downstream target analysis, the successful use of publicly available STAT3 and BATF ChIP-seq data supports the reliability of InTraSeq findings. While the data timepoints do not perfectly align with the InTraSeq experimental conditions, the observed correlations between ChIP targets and InTraSeq data validate the approach. Future studies could generate in-house ChIP-seq data for each timepoint for a more precise alignment with InTraSeq experiments.

This study contributes to the understanding of signaling transduction and gene transcription regulation during T cell activation and cytokine signaling. InTraSeq’s ability to measure protein levels, post-translational modifications of signaling proteins, and transcriptomes at the single-cell level provides novel insights into the interactions between these molecular entities.

## Methods

### PBMC cell preparation

Frozen Human Peripheral Blood Mononuclear Cells (PBMCs) (STEMCELL Technologies #70025) were washed once with 10 mL of Roswell Park Memorial Institute (RPMI) 1640 medium. The cells were spun down at 300 × g for 5 minutes at 4°C, then washed again with 10 mL of Phosphate-Buffered Saline (PBS), then spun down again at 300 × g for 5 minutes at 4°C. The cells were resuspended in 500 uL of PBS prior to be subjected to the InTraSeq protocol (CST #82906). The InTraSeq 3’ Conjugate Antibody Cocktail 1 (CST #48167) was added in the protocol. In the Live, InTraSeq RNA – only, and InTraSeq RNA + ADT experiment, the Live sample represents Frozen PBMCs, the InTraSeq RNA – only sample represents PBMCs that were subjected to the InTraSeq protocol without the addition of the InTraSeq Conjugate Antibody Cocktail 1, and InTraSeq RNA + ADT represents PBMCs that were subjected to the InTraSeq protocol including the InTraSeq Conjugate Antibody Cocktail 1

### Single cell RNA-sequencing and NGS sequencing

After the InTraSeq protocol, all the cells were processed using the 10x Genomics User Guide CG000317 with the aim of recovering 7,000 cells for the PBMC experiments and 6,000 cells for each sample in the Th17 experiment. The samples were sequenced using the NextSeq 2000 and the P2 or P3 kits (100 cycles). Base-calling was performed using NextSeq 1000/2000 Control Software v1.5.0 (RTA v3.10.30). The Supplementary Fig. 1 datasets were sequenced using the NovaSeq 6000 and the S1 and S2 kits (2 × 50cycles). Base-calling was performed using NovaSeq Control Software RTA v3.4.4.

### Flow Cytometry and analysis

Frozen PBMCs (STEMCELL Technologies #70025) were washed twice with Flow Cytometry Antibody Dilution Buffer (CST #13616) then 1 million cells were resuspended in 100 μL of Flow Cytometry Antibody Dilution Buffer and with 2.5 μg of Human BD Fc Block^™^ BD Pharmingen^™^ Human BD Fc Block^™^ (BD Biosciences #564220). After a 10 minute incubation with the Fc block, the cells were washed with the Flow Cytometry Antibody Dilution Buffer (CST #13616) then fixed and permeabilized using the Intracellular Flow Cytometry Kit (Methanol) kit and protocol (CST #13593). The following antibodies were added to the PBMCs at their respective recommended dilution from CST for Flow Cytometry: CD3 (UCHT1) Mouse mAb (PE-Cy7^®^ Conjugate) (CST #62670) to label T cells, CD14 (61D3) Mouse mAb (PE Conjugate) (CST #59896) to label Monocytes, and NCAM1 (CD56) (E7X9M) XP^®^ Rabbit mAb (Alexa Fluor^®^ 647 Conjugate) (CST #50831) to label NK cells, CD19 (Intracellular Domain) (D4V4B) XP^®^ Rabbit mAb (Alexa Fluor^®^ 488 Conjugate) #70418 to label B cells, and with either NFAT1 (D43B1) XP^®^ Rabbit mAb (Pacific Blue^™^ Conjugate) (CST #84791) or with Aiolos (D1C1E) Rabbit mAb (Pacific Blue^™^ Conjugate) (CST #40386). The cells were then washed twice with Flow Cytometry Antibody Dilution Buffer (CST #13616) then analyzed on the BD FACSCelesta^™^ Cell Analyzer flow cytometer.

For the S100A9 flow cytometry experiment, the anti-CD3 and anti-CD14 were replaced with the following respective conjugates CD3 (UCHT1) Mouse mAb (violetFluor^™^ 450 Conjugate) #61347 and CD14 (61D3) Mouse mAb (PE-Cy7^®^ Conjugate) #44947 and the cells were immunostained with S100A9 (D5O6O) Rabbit mAb (PE Conjugate) (CST #91979). Compensation experiments were done appropriately for each color then the data was analyzed using FlowJo 10.10.0.

### Mouse T cell isolation and stimulation

C57BL/6J mice were purchased from Jackson Laboratory (#000664) and housed in Brigham and Women’s Hospital mouse facility. 12-well tissue culture plates were precoated with anti-CD3 (clone: 17A2, 1 μg/ml, BioXCell #BE0002) and anti-CD28 (clone: 37.51,1 μg/ml, BioLegend #102122) in PBS overnight at 4 °C. Mouse splenocytes and lymphocytes were collected and enriched with CD4 (L3T4) microbeads (Miltenyi #130-117-043) followed by ow cytometry sorting of CD4^+^CD25^−^CD62L^hi^CD44^Lo^ for naïve CD4 + T cells. Alternatively, mouse naïve CD4 T cells were also collected with naïve CD4 + T cell kit (Miltenyi #130-104-453). Precoated wells were washed with twice with PBS and seed around 1.1 × 10^6^ of naïve T cells and spun at 500 × g for 5 min for cells to attach. Cells were cultured in T cell expansion serum free media (Gibco, A1048501) and stimulated as follows in addition to anti-CD3/CD28: Th0 (no other cytokines), npTh17 (2 ng/ml TGF-β1, Miltenyi #130-095-067, 20 ng/ml IL-6, R&D systems #406-ML-025), pTh17 (20 ng/ml IL-1β, R&D systems #401-ML-025, 20 ng/ml IL-6, 20 ng/ml IL-23, R&D systems #11269-ML-050), PMA (Sigma-Aldrich #P1585, 5 ng/ml)/Ionomycin (Sigma-Aldrich #I0634, 500 ng/ml). After stimulation for the indicated time, cells were harvested, washed, and undergone downstream InTra-seq, western blotting, or RT-qPCR.

### Western blotting

Stimulated T cells were lysed in RIPA buffer (Thermo Scientific #89900) with protease and phosphatase inhibitors (Thermo Scientific #78444). Whole cell lysates were resolved on 4–12% gels, transferred to PVDF membrane (EMD Millipore #IPFL00010), blocked with 5% non-fat milk (BioRad #1706404) or 1% BSA (Fisher Bioreagents #BP1600), and incubated overnight at 4°C with the following antibodies: Actin (CST #4970, 1:1000), STAT3 (CST #12640, 1:1000), phospho-STAT3 Y705 (CST #9145, 1:1000), phospho-STAT3 S727 (CST #34911), p44/42 (CST #4695, 1:1000), phospho-p44/42 T202/Y204 (CST #4370, 1:1000), S6 (CST #2217, 1:1000), phospho-S6 S235/236 (CST #2211, 1:1000), NF-κB p65 (CST #8242, 1:1000), phospho- NF-κB p65 S536 (CST #3033, 1:1000). Membranes were then washed with 1 × TBST (CST #9997S), blotted with HRP-linked secondary antibody (CST #7074) for 1 hour at room temperature.

### RT-qPCR

RNAs from stimulated cells were isolated with TRIzol reagent (Invitrogen #15596018) per manufacturer’s instructions and reverse transcribed using Superscript IV VILO mastermix (Invitrogen #11756500). Quantitative PCR was done using SYBR green master mix (Thermo Scientific #A25742) with following primers (from 5’ to 3’): Satb1_RT_F: aacactcgggccatctcatg, Satb1_RT_R: tacaaattccgcgtgctcct, Pim1_RT_F: ccgaggattcttctggcagg, Pim1_RT_R: atgagtttgatttcgccgcg, Lif_RT_F: ggtggagctgtatcggatgg, Lif_RT_R: aggcccctcatgacgtctat, Cd200_RT_F: gccatctctccacctacagc, Cd200_RT_R: gtgcagcgcctttctttcat, Gimap5_RT_F: aggagtgtgggaggaggtac, Gimap5_RT_R: gagccctcacactcctgttc, Btla_RT_F: caccaatgcctcaggaccat, Btla_RT_R: ttcagaaagcagagcaggca, Maf_RT_F: gaacaattccgacctgccca, Maf_RT_R: ctgatgatgcggtcggtctc.

### Preprocessing of single-cell RNA-seq and InTraSeq data

InTraSeq data were generated and analyzed independently for multiple experiments. The human PBMCs dataset 1 (Fig. 1, Figure S1A-C, Fig. 2, Figure S2) was preprocessed using 10x Genomics Cell Ranger v7.1.0^42^. Reads were aligned to the Human GRCh38 reference genome (10x Genomics Cell Ranger 2020-A version based on GENCODE v32/Ensembl 98). Default parameters were used unless otherwise noted. To make a fair comparison of the quality of RNA data generated under different protocols, the cells were subsetted and the aligned reads were downsampled. First, 7,000 cells with the highest total number of UMIs were retained to match the number of cells targeted to recover. Next, the aligned reads in the Live and the InTraSeq RNA-only samples were downsampled to match the average aligned reads per cell in the InTraSeq RNA + ADT sample. The aligned read counts were obtained from the “molecule_info.h5” le generated by 10x Genomics Cell Ranger. The new UMI count matrices were then generated based on the non-zero reads after downsampling. Subsequently, low-quality cell profiles were excluded if they fulfilled one or more of the following criteria: (i) the number of genes expressed were in the lowest 1 percentile or the highest 10 percentile, (ii) the total number of UMIs were in the lowest 1 percentile or the highest 10 percentile, (3) the proportion of mitochondrial RNA UMIs were in the highest 10 (Live) or 1 (InTraSeq RNA-only and InTraSeq RNA + ADT) percentile.

The human PBMCs dataset 2 (Figure S1D) was preprocessed using 10x Genomics Cell Ranger v6.1.2 with the same reference genome (Human GRCh38). The human PBMCs dataset 3 and 4 (Figure S1E and F) were preprocessed using 10x Genomics Cell Ranger v7.0.0 with the same reference genome (Human GRCh38). Low-quality cell profiles were excluded if they fulfilled one or more of the following criteria: (i) the number of genes expressed were in the lowest 10 percentile or the highest 10 percentile, (ii) the total number of UMIs were in the lowest 10 percentile or the highest 10 percentile, (3) the proportion of mitochondrial RNA UMIs were > = 10%.

The Th17 differentiation dataset was preprocessed using the cellranger_workflow (v2.4.1) in Cumulus^43^, a cloud-based framework for large-scale single-cell sequencing data analysis. In the workflow, data were preprocessed using 10x Genomics Cell Ranger v7.1.0 and reads were mapped to the mouse mm10 reference genome (10x Genomics Cell Ranger 2020-A version based on GENCODE vM23/Ensembl 98). Low-quality cell profiles were excluded if they fulfilled one or more of the following criteria: (i) the number of genes expressed were in the lowest 5 percentile or the highest 5 percentile, (ii) the total number of UMIs were in the lowest 5 percentile or the highest 5 percentile, (3) the proportion of mitochondrial RNA UMIs were in the highest percentile.

### Analysis of InTraSeq data

Seurat v5.1.0^44^ as used for data analysis and visualization. The RNA UMI count data were log-normalized with scale factor of 10,000, and the ADT UMI count data were normalized using the centered log ratio transformation across cells (*the margin* parameter was set to 2). For proteins with post-translational modifications (PTMs) whose corresponding total protein (without PTM) were also measured, the PTM ratios were computed as the ratio of UMI counts between the PTM and the total protein (with pseudo-count of 1 for both), then the centered log ratio transformation was performed across cells to shrink the extreme values.

Principal Component Analysis (PCA) was performed using the RunPCA function. Numbers of PCs for downstream analyses were selected based on elbow plots. DoubletFinder^45^ was applied to detect multiplets in the PBMCs samples. DoubletFinder didn’t detect clear cluster of high-confidence doublets in the PBMCs dataset 1, thus no cells were excluded in this step. In the other PBMCs datasets, cells classi ed as high-confidence doublets by DoubletFinder were excluded from downstream analysis. DoubletFinder was not applied to the Th17 differentiation dataset because the cells within each sample were homogeneous. Different samples were integrated using the IntegrateLayers function with the HarmonyIntegration method, which calls the Harmony integration algorithm^22^ integrative analysis of the RNA and the ADT modalities was performed using the weighted-nearest neighbor (WNN) method implemented in the FindMultiModalNeighbors function. Uniform Manifold Approximation and Projection (UMAP)^46^ was computed using the RunUMAP function on selected PCs and the UMAP cell embeddings were used for visualization.

Unsupervised clustering was performed using the FindNeighbors and the FindClusters function. Specifically, the FindNeighbors function constructs a Shared Nearest Neighbor (SNN) graph in the following three steps: build a K-Nearest Neighbor (KNN) graph in the PC space, calculate the Jaccard index between every cell and its k nearest neighbors, then remove edges with low Jaccard index. Next, the FindClusters function identifies clusters using a modularity maximization algorithm^47^. Clusters were annotated according to their differentially expressed genes, as well as the expression of known cell type and cell state marker genes (and proteins, if available). Differentially expressed genes were identified using the Wilcoxon rank-sum tests implemented in the FindAllMarkers or the FindMarkers function. In the PBMC dataset 1, one cluster of multiplets was identified based on the co-expression of B cell markers (e.g. CD19 and IGHM) and T cell markers (e.g. CD3E), and was excluded from downstream analysis thereafter. In the Th17 differentiation dataset, one cluster primarily consisting of cells at the 0- and 10-minute time points showed expression of interferon stimulated genes. These cells were likely activated CD4 T cells from ow cytometry sorting of CD4^+^CD25^−^CD44^lo^CD62L^hi^ which can be affected by gating and were excluded from downstream analysis.

### STAT3 and BATF pathway analysis

Integrated analysis of the InTraSeq data and public ChIP-seq data^37^ imputed the targets directly regulated by STAT3 or BATF in Th17 differentiation (Fig. 3H–L and Figure S3M-N). First, npTh17-specific STAT3-bound genes were identified from a public STAT3 ChIP-seq dataset where wild type naïve CD4 + T cells were stimulated with Th0 condition for 24h or npTh17 condition for 3, 16 and 48 hours, and *Stat3* knockout naïve CD4 + T cells were stimulated with npTh17 condition for 24 hours. The npTh17-specific STAT3-bound genes were defined as the union of genes bound by STAT3 in wild type cells treated with npTh17 condition at all time points, excluding those bound by STAT3 in wild type cells treated with Th0 condition or in Stat3 knockout cells. Similarly, npTh17-specific BAFT-bound genes were identified by taking the union of its ChIP-seq binding targets in wild type naïve CD4 + T cells stimulated with npTh17 condition (16 and 48 hours), then excluding those bound by BATF in wild type cells stimulated with Th0 condition (48 hours) or in *Batf* knockout cells (treated with npTh17 condition for 48 hours).

To focus on the STAT3 and BATF target genes that were transcriptionally regulated at during early Th17 differentiation and were associated with Th17 pathogenicity, the npTh17-specific STAT3- and BATF-bound gene lists were further intersected with the differentially expressed genes in both npTh17 vs. Th0 and in npTh17 vs. pTh17 conditions. For each pair of conditions, differential expression analysis was performed using the Wilcoxon rank-sum tests (the FindMarkers function of the Seurat package) at every time points, and genes with Benjamini-Hochberg adjusted p-values (FDR)^24^ < 0.05 and |log_2_ fold change| > log_2_(1.5) in at least on time point were considered differentially expressed.

### Cell cycle analysis

Cell cycle phases were classified using the CellCycleScoring function in Seurat. The G2M and S phase signature genes were obtained from the “cc.genes.updated.2019” object^48^ and converted to mouse gene symbols.

A more precise M phase annotation was feasible using the pH3-Ser10 expression level. We assumed that the UMI count of pH3-Ser10 levels in cells that are not at the M phase follow a negative binomial distribution *X* ~ NB(*μ*, *σ*^2^). Since the S phase is the most distinct from the M phase cells temporally, the distribution of pH3-Ser10 UMI counts could be used to approximate the null distribution:

$$X\sim NB\left(\hat{\mu }=\sum_{i=1}^{n}x_{i},\hat{\sigma^{2}}=\frac{1}{n}\sum_{i=1}^{n}{\left(x_{i}-\hat{\mu }\right)}^{2}\right),$$

where *x*_*i*_ is the observed UMI count in S-phase cell *i* and *n* is the total number of S-phase cells in the sample.

The null hypothesis that a cell is in not at the M phase is rejected if the likelihood of obtaining a pH3-Ser10 UMI count the same as or higher than the observed count is smaller than a pre-determined α level. The Benjamini-Hochberg procedure was used to adjust for multiple comparisons, and the cells with adjusted p-values < α = 0.05 are classified as M phase cells. Code for M phase annotation is available at https://github.com/lhuang1/InTraSeq.

### Image design

A paid Biorender (https://www.biorender.com/) subscription was used during the generation of the images and at the time of submission.

## Data Availability

InTraSeq and other sequencing datasets collected in this study were deposited in Zenodo at XXX (will be made available when the paper is in print.)
